# Transcription of *Ehrlichia chaffeensis* Genes Is Accomplished by RNA Polymerase Holoenzyme Containing either Sigma 32 or Sigma 70 

**DOI:** 10.1371/journal.pone.0081780

**Published:** 2013-11-21

**Authors:** Huitao Liu, Tonia Von Ohlen, Chuanmin Cheng, Bonto Faburay, Roman R. Ganta

**Affiliations:** Department of Diagnostic Medicine/Pathobiology, College of Veterinary Medicine, Kansas State University, Manhattan, Kansas, United States of America; Washington State University, United States of America

## Abstract

Bacterial gene transcription is initiated by RNA polymerase containing a sigma factor. To understand gene regulation in *Ehrlichia chaffeensis*, an important tick-transmitted rickettsiae responsible for human monocytic ehrlichiosis, we initiated studies evaluating the transcriptional machinery of several genes of this organism. We mapped the transcription start sites of 10 genes and evaluated promoters of five genes (*groE, dnaK, hup, p28-Omp14* and *p28-Omp19* genes). We report here that the RNA polymerase binding elements of *E. chaffeensis* gene promoters are highly homologous for its only two transcription regulators, sigma 32 and sigma 70, and that gene expression is accomplished by either of the transcription regulators. RNA analysis revealed that although transcripts for both sigma 32 and sigma 70 are upregulated during the early replicative stage, their expression patterns remained similar for the entire replication cycle. We further present evidence demonstrating that the organism’s -35 motifs are essential to transcription initiations. The data suggest that *E. chaffeensis* gene regulation has evolved to support the organism’s growth, possibly to facilitate its intraphagosomal growth. Considering the limited availability of genetic tools, this study offers a novel alternative in defining gene regulation in *E. chaffeensis* and other related intracellular pathogens.

## Introduction

Several *Anaplasmataceae* family pathogens have been identified in recent years as the causative agents of important diseases in people and various vertebrate animals [1-3]. The limited availability of genetic tools to study these primarily obligate intraphagosomal pathogens hampers our understanding of the molecular mechanisms of pathogenesis. *E. chaffeensis*, a member of the *Anaplasmataceae* family, is a tick-transmitted pathogen responsible for an important emerging disease, human monocytic ehrlichiosis (HME) [4,5]. HME is an acute flu-like illness with symptoms including fever, headache, myalgia, anorexia and chills and is frequently accompanied by leukopenia, thrombocytopenia, anemia, and upgraded levels of serum hepatic aminotransferases [3]. This pathogen also infects several vertebrate animals, including white-tailed deer, dogs, goats and coyotes [6-8]. One of the significant features of *E. chaffeensis* infection, like other members of the *Anaplasmataceae* family, is prolonged persistence in vertebrate and tick hosts [9-12]. *E. chaffeensis* may have evolved specific strategies to establish persistent infections so they can successfully complete their lifecycles in dual hosts. Global host-specific differences in the transcription and expressed proteins of *E. chaffeensis* have been reported [13,14]. The host cell-specific differences in gene expression support the hypothesis that *E. chaffeensis* utilizes novel strategies to adapt and persist in both vertebrate and tick hosts, but the exact molecular mechanism of adaptation is unclear.

Gene expression in bacteria is accomplished by regulating transcription by RNA polymerase (RNAP). RNAP activity in bacteria is often regulated by altering transcription from a gene to adapt to different host environments [15,16]. A typical bacterial RNAP consists of a core RNA polymerase and a transcription regulator, a sigma (σ) factor. The core enzyme typically contains four or five different subunits: two α subunits and one each of β and β’ subunits, and some organisms contain a ω subunit [17]. Binding of a sigma factor to a core RNAP (referred to as an RNAP holoenzyme) enables specific recognition of a promoter element and the transcription initiation. Recognition of a specific promoter by RNAP holoenzyme is one of the important mechanisms that regulates gene expression in bacteria [18-20]. The primary housekeeping sigma factor, sigma 70 (σ^70^), in *Escherichia coli* (*E. coli*) and its homologs in other bacteria are shown to control the transcription of most of the genes during exponential growth of bacterial cells. Alternative sigma factors generally regulate transcription triggered by a specific stress environment or during developmental conversions [21]. The number of alternate sigma factors differs in different bacteria; for example, the *E. coli* genome has 7 sigma factors [22], whereas 65 sigma factors are found in *Streptomyces coelicolor* [23]. The *E. chaffeensis* genome, however, has only two sigma factor genes; *rpoD* (the predicted primary housekeeping σ^70^ gene) and *rpoH* (the predicted alternate σ^32^ gene) [24] (GenBank # NC_007799.1). Both σ^32^ and σ^70^ are conserved in most proteobacteria [25].

Transcription from a gene promoter by a σ^32^- or σ^70^-bound RNAP typically involves recognition of and binding to two DNA motifs located upstream from the transcription start site (TSS) [26]. These include the -35 motif, located about 35 bp upstream of the TSS [20], and the -10 motif which is present at about 10 bp upstream of TSS [20]. The conserved -35 region of promoters recognized by σ^32^ transcription regulator in *E. coli* (TTGAAA) is analogous to the -35 motif recognized by its σ^70^ (TTGACA), [22,27]; however, -10 motifs of σ^32^ promoters in *E. coli* (CCATNT) are markedly different from those recognized by σ^70^ (TATAAT) [27,28]. We recently mapped the promoters of two outer membrane protein genes (*p28-Omp14* and *p28-Omp19*) of *E. chaffeensis* that are transcribed by its σ^70^ transcription regulator [29] and reported that the -35 motif is highly homologous to the consensus *E. coli* -35 motif and that it is required to initiate the transcription. Furthermore, we reported that the -10 motifs of *E. chaffeensis* genes are not homologous to the *E. coli* sequences and are also nonessential for the promoter-specific transcription [29].

In the current study, we used molecular approaches to map several gene promoters of *E. chaffeensis*. We present evidence demonstrating that the σ^32^ and σ^70^ binding motifs share extensive homology for genes likely to be transcribed by the two sigma factors and that the promoter-specific transcription is accomplished by either of the transcription regulators.

## Materials and Methods

### Bioinformatics

Promoter sequences upstream to the transcription start sites (experimentally determined) of genes were evaluated to identify -10 and -35 motifs using WCONSENSUS version v5c (http://stormo.wustl.edu/consensus/cgi-bin/Server/Interface/wconsensus.cgi) [30] and WebLogo (weblogo.berkeley.edu) [31,32] programs. Multiple DNA alignments were done using Clustal X version 2.0 with default parameters [33].

### *E. coli* strains and plasmids

*E. coli* strains used in this study were TOP10 (Invitrogen Technologies, Carlsbad, CA), BL21(DE3)pLysS (Novagen, San Diego, CA) and CAG57101[34]. Genetic makeup of CAG57101 is included in Table 1. Several plasmid constructs used in this study were obtained from commercial sources or recombinantly modified from one or more existing plasmids described in the literature. They include pCR2.1 TOPO (Invitrogen Technologies), pET32a (Novagen) and the derivatives of pSAKT32 [35], pQF50K [35] and pMT504 [36]. Genetic makeup details of the original plasmids and their derivatives are included in Table 1 for all plasmids except those obtained from commercial sources. The plasmid pSAKT32, with a p15A origin of replication and containing an ampicillin resistance gene, has *E. coli rpoH* under the control of an IPTG inducible wild-type P_lac_ promoter [34,35]. The *E. coli rpoH* from this plasmid was replaced with the *E. chaffeensis rpoH* (Ech_*rpo*H). Cloning was accomplished by digesting the pSAKT32 with Afl II and Sal I to remove the *E. coli rpoH*, blunt ending with Klenow DNA polymerase (BioLabs, Ipswich, MA), then ligating with the Ech_*rpo*H sequence. Ech_*rpo*H was generated by PCR from genomic DNA using Pfu DNA polymerase (Promega, Madison, WI). The final derived plasmid is referred as the pSAKT32-Ech_rpoH. All *E. chaffeensis rpoH* variants (substitutions within the 4.2 region of *E. chaffeensis* σ^32^) were constructed with a QuickChange Lightning Site-Directed Mutagenesis Kit (Agilent Technologies, La Jolla, CA). The names of the modified pSAKT32-Ech_rpoH are provided in Table 1.

**Table 1:** Bacterial strain and plasmids used in this study.

Name	Description	Reference
***E. coli***		
CAG57101	MG1655 *△lacX74*, P_BAD__*groESL::cat*, *△rpoH::aadA*, Cm^R^, Sp^R^	[34]
**Plasmids**		
pSAKT32	p15A ori, P_lac_, *E. coli rpoH*, *lacI* ^q^, Amp^R^	[35]
pSAKT32-Ech_rpoH	For *E. chaffeensis* σ^32^ expression	This study
pSAKT32-Ech_rpoH_266	σ^32^-E266A in pSAKT32-Ech_rpoH	This study
pSAKT32-Ech_rpoH_267	σ^32^-R267A in pSAKT32-Ech_rpoH	This study
pSAKT32-Ech_rpoH_269	σ^32^-R269A in pSAKT32-Ech_rpoH	This study
pSAKT32-Ech_rpoH_270	σ^32^-Q270A in pSAKT32-Ech_rpoH	This study
pQF50K-groE (*E. coli*)	pMB1 ori,1600 ori, *E. coli groE* promoter, *lacZ*, Kan^R^	[35]
pQF50K-Ech_dnaK	*E. chaffeensis dnaK* promoter in pQF50K	This study
pQF50K-Ech_groE	*E. chaffeensis groE* promoter in pQF50K	This study
pQF50K-Ech_hup	*E. chaffeensis hup* promoter in pQF50K	This study
pQF50K-Ech_dnaK-35del	*E. chaffeensis dnaK* promoter with -35 motif deletion	This study
pQF50K-Ech_hup-35del	*E. chaffeensis hup* promoter with -35 motif deletion	This study
pQF50K-Ech_groE-35del	*E. chaffeensis groE* promoter with -35 motif deletion	This study
pQF50K-Ech_groE-35updel	*E. chaffeensis groE-35updel* in pQF50K	This study
pMT504	Amp^R^; as templates of *in vitro* transcription	[36]
pMT504-p28-Omp19	*E. chaffeensis p28-Omp19* promoter in pMT504	[37]
pMT504-p28-Omp19-R	Reverse orientation *E. chaffeensis P28-Omp19* promoter	[37]
pMT504-p28-Omp19-35	*E. chaffeensis p28-Omp19* promoter with -35 motif deletion	This study
pMT504-p28-Omp14	*E. chaffeensis p28-Omp14* promoter in pMT504	[37]
pMT504-p28-Omp14-R	Reverse orientation *E. chaffeensis P28-Omp14* promoter	[37]
pMT504-p28-Omp14-35	*E. chaffeensis p28-Omp14* promoter with -35 motif deletion	This study
pMT504-Ech_dnaK	*E. chaffeensis dnaK* promoter in pMT504	This study
pMT504-Ech_dnaK-R	Reverse orientation *E. chaffeensis dnaK* promoter	This study
pMT504-Ech_groE	*E. chaffeensis groE* promoter in pMT504	This study
pMT504-Ech_groE-R	Reverse orientation *E. chaffeensis groE* promoter	This study
pMT504-Ech_hup	*E. chaffeensis hup* promoter in pMT504	This study
pMT504-Ech_hup-R	Reverse orientation *E. chaffeensis hup* promoter	This study
pET32-Ech_rpoH	For overexpression of *E. chaffeensis* σ^32^	This study
pET32-Ech_rpoD	For overexpression of *E. chaffeensis* σ^70^	[37]

The plasmid pQF50K, with a pMB1 origin of replication and carrying a kanamycin resistance gene cassette, contains the β-galactosidase coding sequence (*lacZ*) driven by *E. coli groE* promoter for use in assessing the promoter’s function [35]. It was modified to replace with *E. chaffeensis* promoters of *groE, hup* or *dnaK* genes in front of the *lacZ* coding sequence. The full length *groE* promoter of *E. chaffeensis* or the promoter lacking the -35 motif or the promoter lacking the -35 motif and the entire sequence upstream to it were amplified using the gene-specific primers and the organism’s genomic DNA as the template. Similarly, *hup* and *dnaK* promoter segments or those lacking -35 motifs were amplified with Sph I and Xba I restriction enzyme sites engineered to facilitate directional cloning. The promoter segments were cloned upstream to the *lacZ* coding sequences after deleting the *E. coli groE* promoter. The promoter derivatives with deletion of -35 motifs were constructed in the pQF50K-Ech_promoter plasmids by using a QuickChange Lightning Site-Directed Mutagenesis Kit (Agilent Technologies). The names of all engineered plasmids are listed in (Table 1).

The pET32a plasmid vector (Novagen) encoding *E. chaffeensis* σ^32^ and σ^70^ were prepared and used to prepare purified recombinant proteins. The cloning and purification of *E. chaffeensis* σ^70^ is reported earlier [37]. *E. chaffeensis* σ^32^ was cloned similarly into pET32a vector and was utilized to prepare recombinant protein as in [37]. 

Full-length *E. chaffeensis* promoter segments of *dnaK*, *hup* and *groE* were also cloned into the plasmid pMT504 at the EcoR V site for use in the *in vitro* transcription analysis (described below). The promoter inserts were also cloned in opposite orientation to serve as negative controls to demonstrate promoter-specific *in vitro* transcription. The pMT504 has a G-less cassette to serve as the transcription template [36]. Promoter segments of *p28-Omp14* and *p28-Omp19* genes were closed in pMT504 for *in vitro* transcription analysis and reported earlier [37]. pMT504 constructs lacking the -35 motifs for *p28-Omp14* and *p28-Omp19* were generated by PCR cloning strategy using the previously prepared -35 deletion plasmids in pBlueTOPO [29] as templates. The predicted lengths of transcripts for the promoter segments *groE*, *dnaK, hup*, of *p28-Omp14* and *p28-Omp19* in pMT504 plasmid are 155, 156, 172, 162 and 162 nucleotides, respectively (as per the defined transcription start sites). Integrity of all cloned segments in the plasmid constructs described here was confirmed by automated DNA sequencing analysis using Beckman coulter CEQ 8000 Genetic Analysis System (Beckman Coulter, CA). (All primers used for various plasmid manipulations were described in Table S1.)

### *E. coli* growth conditions

CAG57101 *E. coli* strain alone or with the recombinant plasmids were grown as described earlier [34]. Briefly, cultures were grown at 30°C in Luria-Bertani (LB) medium with chloramphenicol (30 μg/ml) and spectinomycin (50 μg/ml) to support the strain’s growth. Ampicillin (100 μg/ml) and kanamycin (50 μg/ml) were added to maintain the pSAKT32-derived plasmids and pQF50K-derived plasmids, respectively.

### *E. chaffeensis* RNA extraction for use in primer extension analysis and 5’RACE

Total RNA was extracted from *E. chaffeensis* organisms recovered from *in vitro* cultures in the canine macrophage cell line, DH82, (80-100% infection) using a Tri Reagent RNA isolation kit (Sigma-Aldrich, St Louis, MO) [38,39]. Residual genomic DNA in RNA preparations was eliminated using a Turbo DNA-free kit (Invitrogen Technologies). Ribosomal RNA (rRNA) (both bacterial and eukaryotic) was digested using the Terminator 5’-Phosphate-Dependent Exonuclease (Epicentre), which selectively removes 5’ monophosphate containing RNAs (rRNA). Bacterial mRNA was further enriched with a MICROB*Enrich* kit (Invitrogen Technologies). Concentration of purified RNA samples was determined by the NanoDrop method (Thermo Scientific, Wilmington, DE).

### Primer extension analysis

Primer extension (PE) analysis was carried out using a Primer Extension System AMV Reverse Transcriptase kit (Promega). Briefly, transcript-specific oligonucleotides were synthesized and end-labeled with [γ-^32^p] ATP and T4 polynucleotide kinase (Promega) (primers were listed in Table S1). One picomole of each of the end-labeled primers was annealed to *E. chaffeensis* RNA (~10 μg), and a reverse transcription reaction was performed with one unit of AMV reverse transcriptase at 42°C for 30 min. The reaction products were electrophoresed on a 6% polyacrylamide gel containing 7 M urea, transferred to a Whatman paper, dried and exposed to an X-ray film. Plasmid DNA templates containing the respective gene segments were used in a Sanger’s DNA sequence reaction to generate the DNA sequence ladders to identify the transcription start sites (TSS) using a Thermo Sequenase Dye Primer Manual Cycle Sequencing Kit (USB, Cleveland, OH). The PE products were detected after developing the film with a Konica film processor (Konica, Wayne, NJ). 

### 5’ Rapid Amplification of cDNA Ends (5’RACE)

5’RACE experiments were performed on *E. chaffeensis* RNA (1 µg) to map the TSSs using a 5’ RACE kit version 2.0 (Invitrogen Technologies). The final products were resolved in a 1% agarose gel; the major amplicons were gel isolated and used to perform DNA sequencing analysis. (5’RACE primers are listed in Table S1.)

### *In vitro* transcription assays

*In vitro* transcription reactions were performed in 10 μl reaction mixture containing 0.13 picomoles each of the supercoiled plasmid DNAs as templates and using RNAP holoenzymes containing either recombinant *E. chaffeensis* σ^32^ or σ^70^ as in [37]. The holoenzymes were prepared by incubating 0.5 μl of 1:10 diluted stock of *E. coli* core enzyme (Epicentre, Madison, WI) mixed with 10 fold molar excess of purified recombinant *E. chaffeensis* σ^32^ or σ^70^ on ice for 30 min. The transcription reactions were incubated at 37°C for 20 min, and the reactions were terminated by adding 7 μl of stop solution (95% formamide, 20 mM EDTA, 0.05% bromophenol blue and 0.05% xylene cyanol). Six microliters each of the samples were electrophoresed on a 6% polyacrylamide sequencing gel with 7 M urea, then gels were transferred to a Whatman paper, dried and transcripts were visualized by exposing an X-ray film to the gels. Control reactions included only *E. coli* core enzyme or recombinant σ^70^ or σ^32^ in the absence of core enzyme. Additional controls included the use of promoter constructs prepared in reverse orientation or promoters lacking -35 motifs (promoters of *p28-Omp14* and *p28-Omp19* genes). 

### Electrophoretic mobility shift assays (EMSAs)

EMSAs were performed with a LightShift Chemiluminescent EMSA kit (Pierce Biotechnology, Rockford, IL) with minor modifications. Briefly, biotin labeled probes were prepared by PCR from *E. chaffeensis* genomic DNA as the template and using probe-specific oligonucleotide primers (one primer contained biotin at the 5’ end; the primers were listed in Table S1). The amplicons were purified using Qiagen DNA columns (QIAGEN, Valencia, CA). Cold competitor probes were produced in the same manner, except that neither of the primers used in the PCRs contained biotin tags. All competitor DNAs were used in 100-fold molar excess compared with the labeled probes. DNA–protein binding reactions were carried out at 30°C for 25 min in 20 µl volume containing 1x binding buffer [50 mM Tris-acetate (pH 8.0), 50 mM potassium acetate, 8.1 mM magnesium acetate, 27 mM ammonium acetate, 100 μg/ml BSA and 5% glycerol], 50 μg/ml poly dI-dC and 20 fmol each of a probe and RNAP holoenzyme. The holoenzyme was assembled with 0.25 μl *E. coli* RNAP core enzyme (Epicentre) and 10-fold molar excess of *E. chaffeensis* σ^32^ or σ^70^ relative to the *E. coli* RNAP core enzyme and incubated on ice for 30 min prior to use in the EMSAs. Control reactions included only *E. coli* core enzyme or recombinant σ^70^ or σ^32^ in the absence of core enzyme or used an open reading sequence probe of *dnaK* (*dnaK-*ORF). The reactions were stopped by adding 5 μl of gel loading buffer and electrophoresed in 4% native polyacrylamide gel in 0.5X TBE buffer at 80 V for 1.5 h, then the DNA and DNA–bound proteins were transformed to a nylon membrane by electrophoretic transfer. Biotinylated DNA fragments were detected by the chemiluminescence method (Pierce Biotechnology).

### β-Galactosidase assays

Overnight *E. coli* cultures of CAG57101 were diluted 1:100 into a fresh medium containing appropriate antibiotics, grown to 0.6–0.8 optical density, and induced with 1 mM IPTG for 3 h before harvesting. *E. coli* lysates were then prepared and used to measure β-galactosidase activity using a β-gal assay kit (Invitrogen Technologies). IPTG-non induced cultures were used to serve as the controls. The experiment was performed three times with three independently grown cultures, and specific activity of β-galactosidase was calculated using the formula (specific activity = nmoles ONPG hydrolyzed/ min/mg protein).

### Determination of the *E. chaffeensis* rpoD and rpoH Gene Expression by TaqMan-based Quantitative RT-PCR

DH82 macrophage culture-derived *E. chaffeensis* organisms from a nearly 100% infected culture flask was purified and used to infect flasks containing naïve DH82 cultures. Total RNA was isolated from cell cultures several times post-inoculation (6 h to 84 h). Total RNA was also recovered from uninfected cultures to serve as a zero time point and also to serve as the negative control for the analysis. Total RNA was recovered using a Tri Reagent RNA isolation kit. The residual genomic DNA was eliminated from RNA preparations using a Turbo DNA-free kit (Invitrogen Technologies). Gene-specific primers targeting the *rpoD* or *rpoH* gene transcripts (Table S1) or the 16S rRNA (primers and probes were described earlier [40]) were used in real-time RT-PCR analysis using cDNAs as the templates and target-specific TaqMan probes. Total cDNA was synthesized from each RNA sample after annealing random oligonucleotides and by performing reverse transcription using a SuperScript III First Strand cDNA Synthesis System kit (Invitrogen Technologies). Concentrations of cDNAs were adjusted based on the initial PCR analysis targeting the 16S rRNA, and equal amounts of cDNAs were used to perform the TaqMan-based real-time PCR assays as in [40]. The Ct values were normalized based on 16S rRNA transcripts and converted to fold change relative to zero time point RNA [41].

### Statistical analysis

Statistical analyses were performed using Student's *t*-test, and a *P*-value <0.01 was considered significant.

## Results

### Mapping the transcription start sites (TSS) and locating the RNA polymerase binding motifs of *E. chaffeensis* genes

**Figure 1:**
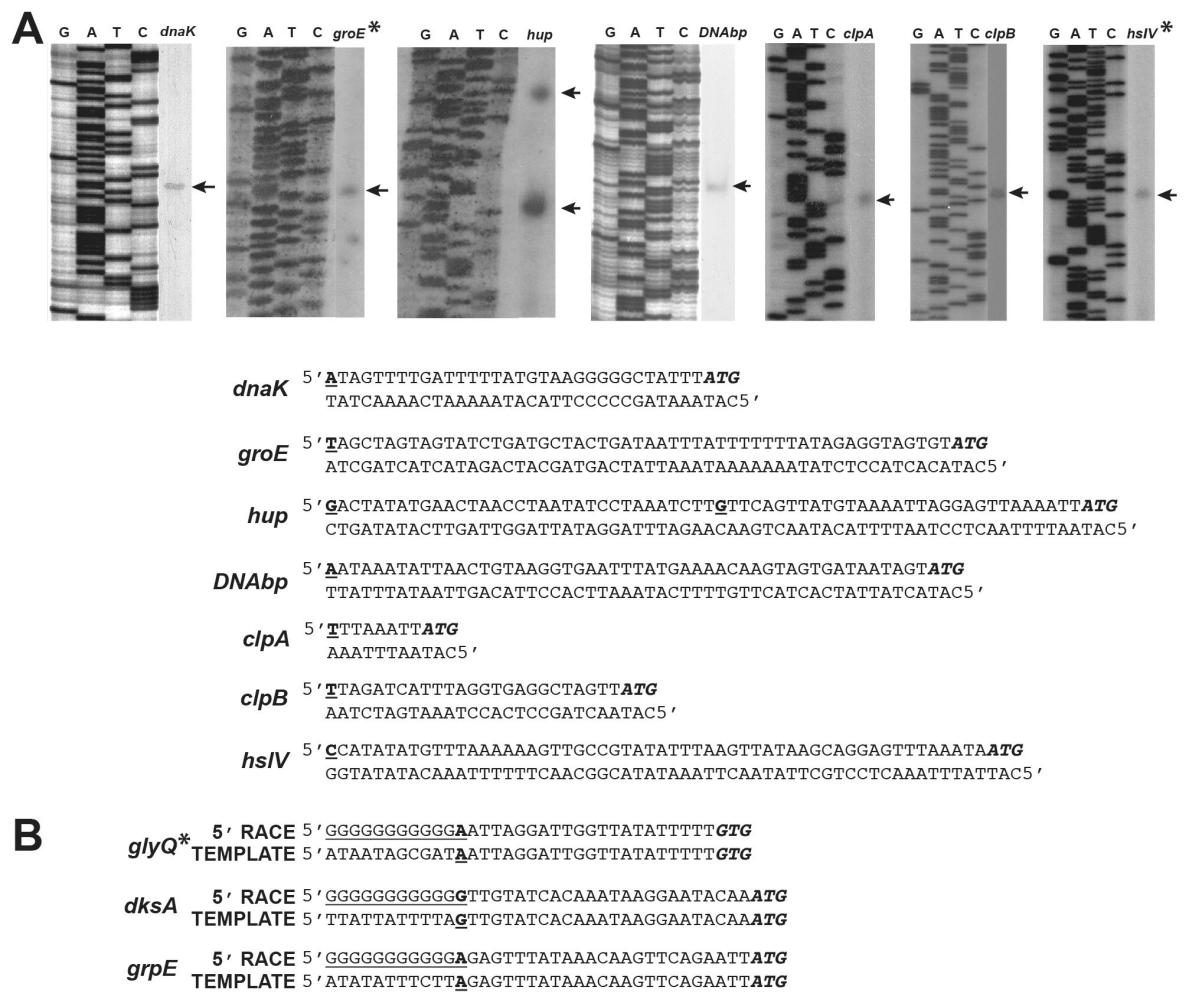
Mapping Transcription Start Sites (TSS) of *E. chaffeensis* genes by primer extension (PE) and 5’ RACE. A) The primer extended products resolved on sequencing gels with TSS identified for the genes *dnaK, hup, DNAbp* (DNA binding protein gene), *clpA, clpB*, operons of groE* (*groES* and groEL genes), and hsIV* (*hslV* and *hslU* genes). The location of the TSS for each gene was identified by comparing the Sanger’s DNA sequencing runs generated with the same primers used for the PE reactions, but using plasmid DNAs containing the respective gene segments. All genes had one TSS with the exception of hup, which contained two TSS. [The location of the TSS established from the PE results for all 7 genes (bold and underlined text) relative to the initiation codon of each gene were presented under gel data.] B) 5’RACE data identifying the TSS of the genes *dksA* and *grpE*, and the operon glyQ* (*glyQ, glyS* and *dnaJ* genes). Sequences generated from the 5’RACE products are compared with the sequences generated with DNA templates and are shown for each gene relative to the initiation codon. TSS are identified with bold and underlined text. The underlined G rich tails are added upstream to TSS during the 5’RACE reaction.

**Figure 2:**
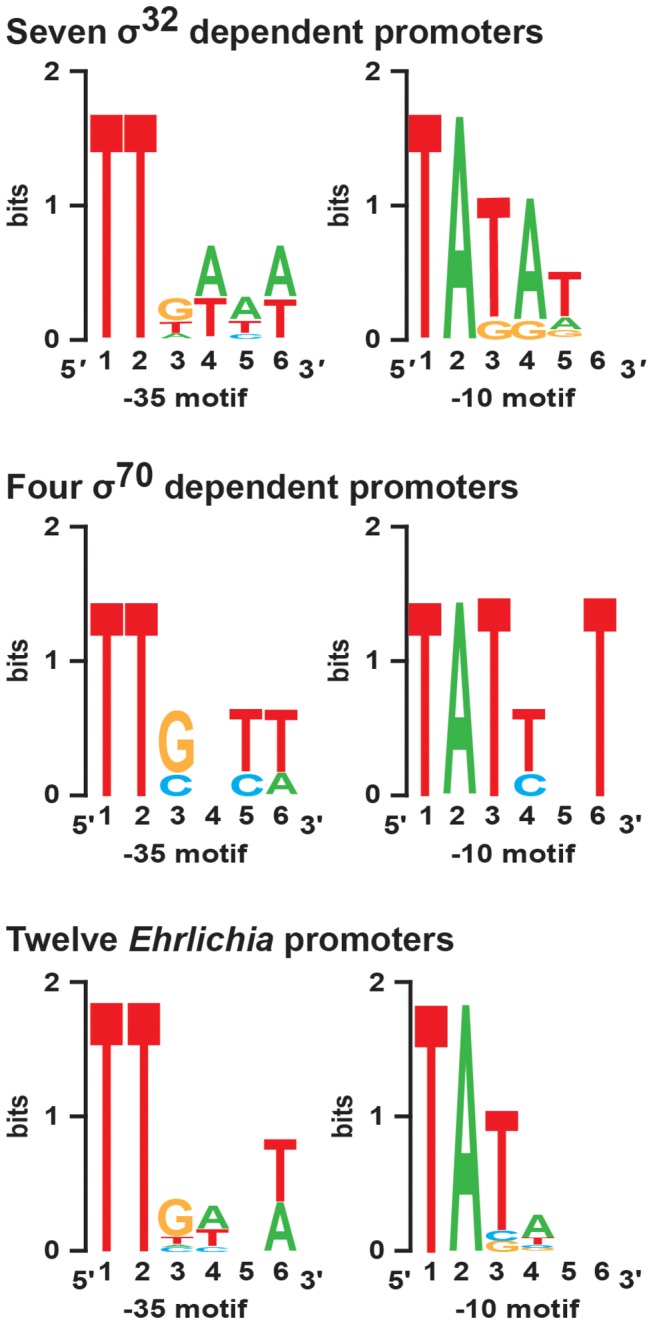
RNAP binding motifs -35 and -10 of *E. chaffeensis* genes. RNAP binding motifs, -35 and -10, are identified for the 12 *E. chaffeensis* genes for which TSS were mapped (listed in Tables 2). The upper panel has the consensus motifs for the σ^32^ dependent gene promoters; the middle panel includes the σ^70^-dependent promoters and the lower panel includes the consensus motifs for all 12 genes assessed.

The *E. chaffeensis* genome contains only two genes encoding for sigma factors (GenBank # NC_007799.1) [24]; the primary housekeeping sigma factor, σ^70^, gene (*rpoD*) (genome locus_tag # Ech_0760) and a homolog of the most conserved alternate sigma factor, σ^32^, gene (*rpoH*) (genome locus_tag # Ech_0655). Our independent homology search analysis further confirmed that these two genes showed the greatest homology with *E. coli* σ^70^ and σ^32^, respectively. TSS for 12 genes of *E. chaffeensis* were mapped by primer extension or 5’RACE analysis; 10 genes were mapped in the current project (Figure 1), and two were mapped previously [29]. These included 7 genes likely recognized by the σ^32^ and four likely recognized by σ^70^, because their homologs in *E. coli* were previously mapped as σ^32^ or σ^70^ genes [22,27,42]. Sequences upstream from the TSS of all 12 genes were assessed to locate the -35 and -10 motifs (RNAP binding motifs) (Table 2), and consensus sequences were generated (Figure 2). The consensus -35 and -10 motifs for the 7 *E. chaffeensis* σ^32^ genes were TTGAAA and TATATN, respectively, and consensus sequences for the four σ^70^ genes were TTGNTT and TATTNT, respectively. The predicted RNAP binding motifs for all 12 genes assessed were TTGWNW and TATANN. Independent of genes likely recognized by σ^32^ or σ^70^, the predicted RNAP binding motifs for all 12 genes were similar in that they conserved three nucleotides (TTG) at the 5’ end of the -35 motifs and three nucleotides (TAT) at the 5’ end of the -10 motifs. 

**Table 2:** The TSS and -35 and -10 motifs of *E. chaffeensis* genes.

Genes/operons*	Locus-tag	-35	-10	TSS^$^ mapped by
**σ^32^ promoters**				
*dnaK*	ECH_0471	TTGTAAtcttatgatttggttatTATATCtgtgattA		PE
*groE	ECH_0364-0365	TTGAAAttagatttcttgatatTATATAtgatctT		PE
*clpA*	ECH_0567	TTATTTtcaacttatttttaattctTAGGTGgtagagtttT		PE
*hslV	ECH_0996-0997	TTTACAtttacttctcaagtggTATAGAtaaagC		PE
*clpB*	ECH_0367	TTTAATttttataaataatgttTATATGtaagT		PE
*glyQ	ECH_0023-0025	TTGTATaacttcatattattcttcTATAATagcgatA		5’ RACE
*grpE*	ECH_0168	TTGATAttttttcataatgttacTATATAtttcttA		5’ RACE
**σ^70^ promoters**				
*p28-Omp19**^+^***	ECH_1143	TTGCTTttatatgacacttctacTATTGTtaA		PE
*p28-Omp14**^+^***	ECH_1136	TTGCTTttctttatttctttcatTATTCTtaA		PE
*hup*	ECH_0804	TTGACTatatgaactaacctaaTATCCTaaatcttG		PE
*dksA	ECH_0064	TTCTTAatcaactatatttaaatTATTATtttaG		5’ RACE
**Unidentified promoter**				
*DNAbp*	ECH_1013	TTGAGTttatgtactttaaattgTACAAAaatgtA		PE

^$^Transcription start sites (TSS) mapped by primer extension (PE) or 5’ RACE were identified as underlined and italic text (last nucleotide in each sequence). **^*+*^**TSS of *p28-Omp14* and *p28-Omp19* were mapped previously [29]. *Operons as listed in Figure 1. -35 and -10 (underlined text) motifs identified for each gene.

### *In vitro* transcription for *dnaK*, *groE*, *hup*, *p28-Omp14* and *p28-Omp19* promoters using recombinant *E. chaffeensis* σ^32^ and σ^70^

**Figure 3:**
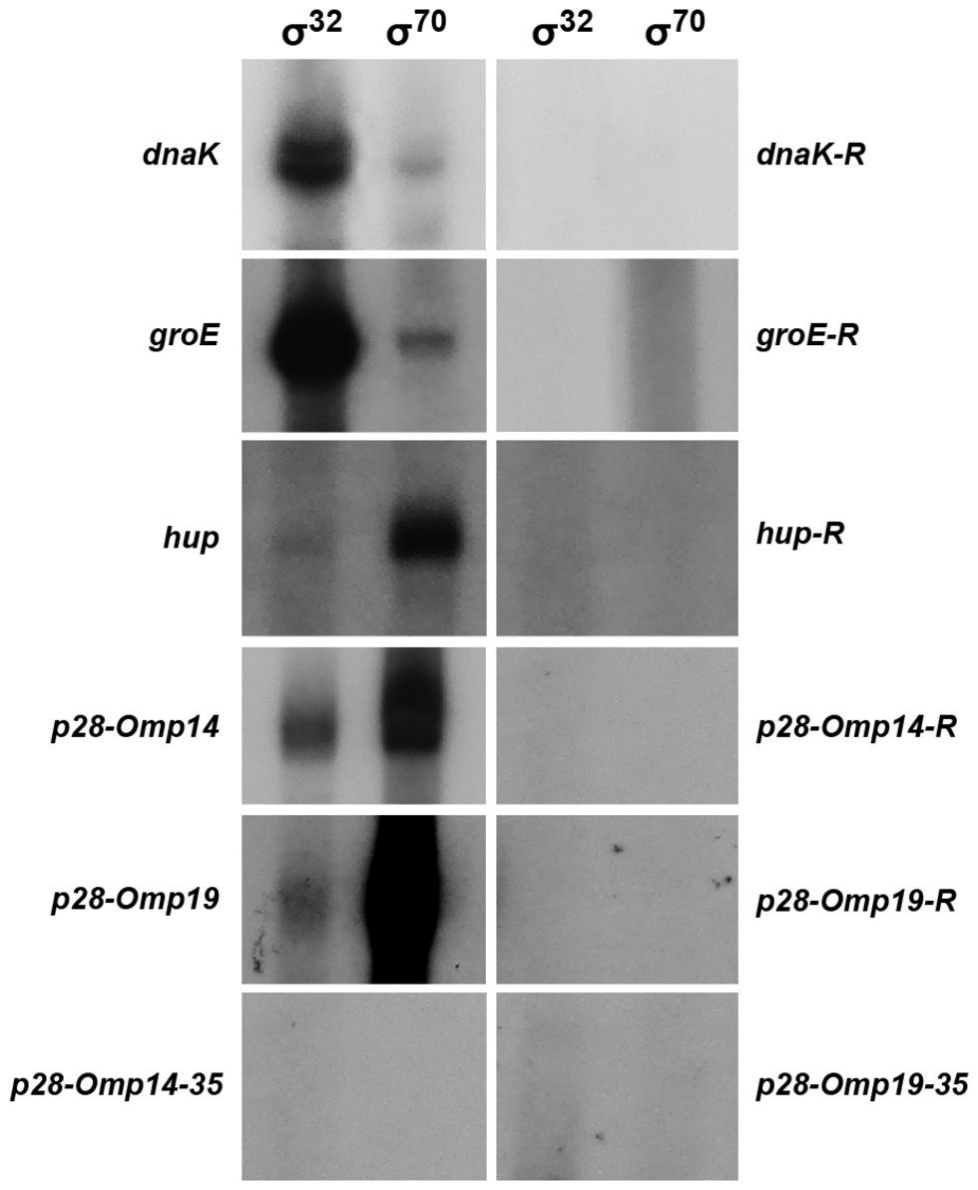
*In* *vitro* transcription analysis of *E. chaffeensis* genes *dnaK*, *groE, hup*, p28-*Omp14* and p28-*Omp19* promoters. *In* *vitro* transcription analysis was performed using RNAP holoenzyme containing *E. chaffeensis* recombinant σ^32^ or σ^70^. The promoter segments of *E. chaffeensis* genes *dnaK*, *groE, hup*, p28-*Omp14* and p28-*Omp19* cloned upstream to the G-less cassette in pMT504 plasmid vector in the correct or reverse orientation were used in the assays with reconstituted RNAP containing *E. chaffeensis* recombinant σ^32^ or σ^70^. (Reverse orientation constructs were identified in the Figure as *dnaK-R, groE-R, hup*-R, p28-*Omp14-R* and p28-*Omp19-R*.) The p28-*Omp14* and p28-*Omp19* promoter constructs having -35 motif deletions (p28-*Omp14-35* and p28-*Omp19-35*) were also prepared and used in the *in* *vitro* transcription assays. The abundance of the transcripts for each gene in the presence of σ^32^ or σ^70^ is captured from the ^32^P incorporation in the RNA. As reported earlier [37], assays performed with RNAP core enzyme alone or with purified σ^32^ or σ^70^ did not yield any transcripts (not shown).

The high degree of conservation and similarities in the RNAP binding motifs of the predicted σ^32^ and σ^70^ genes in *E. chaffeensis* is striking and suggests that the gene promoters of the organism are likely recognized by both the sigma factors to initiate the transcription. To test this hypothesis, we expressed and purified *E. chaffeensis* σ^32^ (current study) and σ^70^ [37] and used the recombinant proteins to perform *in vitro* transcription assays as described earlier using *E. coli* core enzyme to reconstitute the RNAP complex [37]. The promoter sequences of *dnaK, groE* and *hup* were cloned into the G-less cassette plasmid [36] to serve as transcription templates. We previously created similar promoter constructs for the *p28-Omp14* and *p28-Omp19* genes of *E. chaffeensis* [37]. *In vitro* transcription from the RNAP with *E. chaffeensis* recombinant σ^32^ or σ^70^ resulted in generation of the predicted size transcripts for all five gene promoters assessed in the experiment: *dnaK*, *groE*, *hup*, *p28-Omp14* and *p28-Omp19* (Figure 3). Similar to our previous observations [37], the recombinant σ^32^ or σ^70^ alone or *E. coli* core enzyme in the absence of the recombinant sigma factor proteins did not result in generation of *in vitro* transcripts (data not shown). Likewise, all five promoter segments cloned in reverse orientation or promoters lacking -35 motifs (*p28-Omp14* and *p28-Omp19*) did not result in the generation of *in vitro* transcripts (Figure 3). Because -35 motifs were found to be critical for the promoter function of *p28-Omp14* and *p28-Omp19* [29], these promoters lacking the -35 motifs served as additional negative controls for the *in vitro* analysis. *In vitro* transcripts for the promoters in correct orientation were more abundant with the recombinant σ^32^ for *dnaK* and *groE* than those made with the recombinant σ^70^, whereas the opposite is true for the promoters of *hup, p28-Omp14* and *p28-Omp19*. Importantly, all five gene promoters were recognized by the RNAP complexes made with either σ^32^ or σ^70^. *In vitro* transcription analysis data for the two previously mapped σ^70^ promoters of outer membrane protein genes, *p28-Omp14* and *p28-Omp19*, were consistent with our prior results [37] and demonstrated that these promoters were also recognized by the σ^32^. The abundance of transcripts, however, was higher with the RNAP containing recombinant σ^70^ compared to those observed for σ^32^.

### Promoter activity of *dnaK*, *groE* and *hup* assessed in *E. coli* mutant for σ^32^ following induced expression of *E. chaffeensis* σ^32^

**Figure 4:**
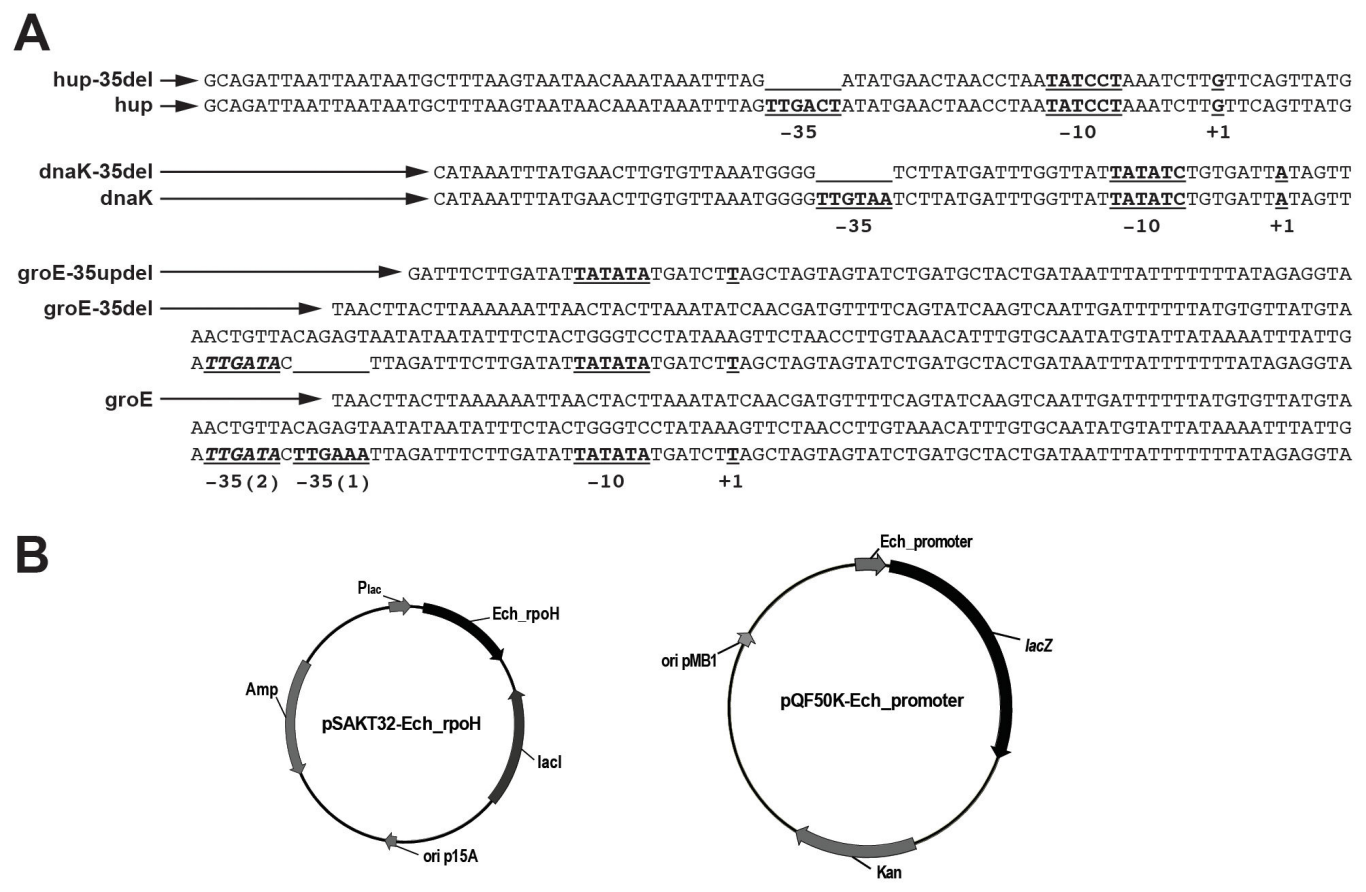
Promoter sequences and the plasmids used for the *in* *vivo* assay system for defining the promoter activities of *E. chaffeensis* genes in *E. coli*. A) Sequences of promoter segments of genes *hup, dnaK* and *groE* (wild-type; hup, dnaK and groE or with -35 deletions; hup-35del, dnaK-35del and groE-35del) used for the *in* *vivo* assays were presented. The groE-35updel segment had a deletion lacking both the -35 motif and the entire sequence upstream from it. The transcription start sites (identified as +1), -35 and -10 motifs of the promoters were identified in the sequences as the bold and underlined text. The second predicted -35 sequence for *groE* promoter was identified as the bold, underlined and italics text. B) Illustrations of the two plasmids with distinct origins of replication used in the *in* *vivo* promoter mapping assays. Plasmid pSAKT32-Ech_rpoH contained either a wild-type *E. chaffeensis* *rpoH* gene sequence or mutant forms with mutations engineered in the region 4.2 of *E. chaffeensis* σ^32^. The pQF50K-Ech_promoter plasmids with promoter sequences described in panel A were cloned upstream to the *lacZ* gene coding sequence to drive the expression of *lacZ* gene.

**Figure 5:**
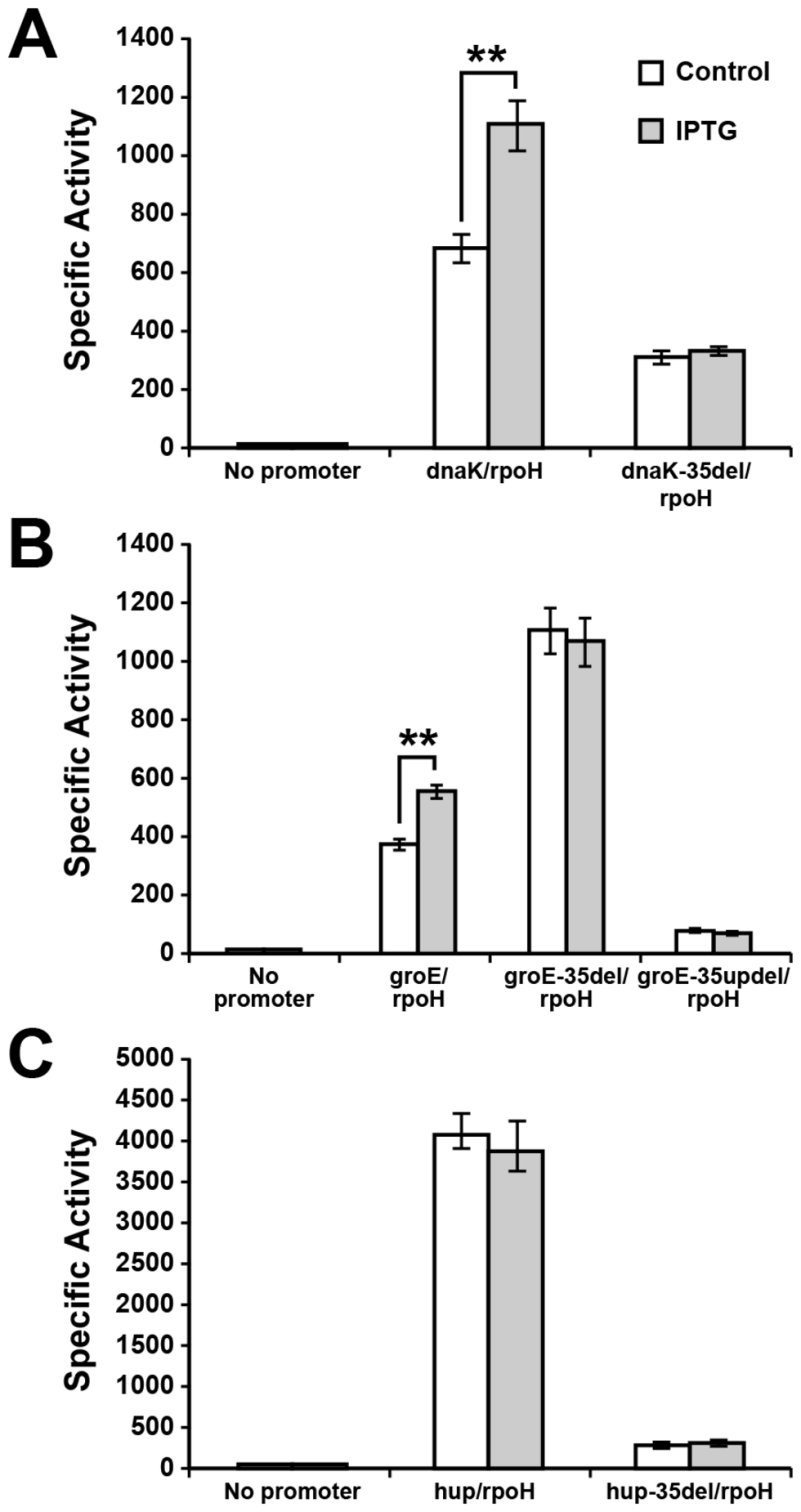
*E. chaffeensis* promoter activities (A, *dnaK*; B, *groE*; and C, *hup*) assessed in *E. coli* by measuring the β-galactosidase expression. The β-galactosidase expression driven by *E. chaffeensis* promoters from the wild-type promoters (*dnaK, groE* or *hup*), promoters containing -35 motif deletion (*dnaK-35del*, *groE-35del* or hup*-35del*) or *groE* promoter having complete deletion from -35 to the entire upstream sequence (groE-35updel) were measured in the CAG57101strain of *E. coli* before or after the induced expression of *E. chaffeensis* *rpoH*. The CAG57101 strain contained either promoterless pQF50K (control) or one of the pQF50K-Ech_promoter plasmids together with the pSAKT32-Ech_rpoH plasmid (described in Figure 4). Three independent experiments were performed; the error bars indicate standard deviation. Significant changes in the β-galactosidase activity were identified with double asterisks where the P values were <0.01.

*In vitro* transcription analysis demonstrated that the *E. chaffeensis* transcription regulators, σ^32^ and σ^70^, were not exclusive in their recognition of the promoters, although their promoter recognition specificities differed. To further define the promoter activities of *E. chaffeensis*, we developed an assay in an *E. coli* strain, CAG57101, in which its chromosomal σ^32^ was inactivated [34] and *E. chaffeensis* σ^32^ protein was expressed from a plasmid under the control of IPTG-inducible P_lac_ promoter. The *E. coli* strain was transformed with a second recombinant plasmid containing *E. chaffeensis* promoter segments of genes *dnaK*, *groE* or *hup*, which were cloned upstream to the promoterless β-galactosidase gene coding sequence. We reasoned that the *E. coli* RNAP core enzyme forms a functional holoenzyme complex with *E. chaffeensis* σ^32^ *in vivo* (similar to *in vitro* studies) when it is expressed. This experiment is similar to studies reported as characterizing the RNAP function of other bacterial organisms [34,35,43]. (Promoter segments and plasmids used for this assay system were described in Figure 4). Induction of the *E. chaffeensis* σ^32^ expression in *E. coli* resulted in the increase of β-galactosidase expression for two gene promoters (*dnaK* and *groE*) (Figure 5). The increase in β-galactosidase expression is significant and is about 60% and 45% above the background level observed for the *dnaK* and *groE* promoters, respectively. The β-galactosidase activity for the *hup* promoter remained unchanged in the presence or absence of *E. chaffeensis* σ^32^ expression. In controls where *E. chaffeensis* σ^32^ expression was not induced, β-galactosidase was also made from the *dnaK* and *groE* promoters; however, the enzyme activity was considerably low compared with that observed for the *hup* promoter which had about 6-10 times higher β-galactosidase activity compared with that observed for *dnaK* and *groE* promoters. Because the *in vitro* transcription analysis (described above) revealed that the *hup* is predominantly a σ^70^ specific promoter (Figure 3), we reasoned that the high *hup* promoter activity in *E. coli* is due to its recognition by the *E. coli* σ^70^. The transcription increase for *dnaK* and *groE* promoters in the *E. coli* strain following inducing the *E. chaffeensis* σ^32^ is consistent with the observations made from *in vitro* transcription analysis, thus validating that the *dnaK* and *groE* promoters have higher affinity for the σ^32^, whereas the *hup* promoter was predominantly recognized by the σ^70^. We reasoned that the low level promoter activity observed for *dnaK* and *groE* promoters in the absence of *E. chaffeensis* σ^32^ is also the result of the promoters’ recognition by the *E. coli* σ^70^. This observation is also consistent with the prior study demonstrating that *E. coli* σ^70^-bound RNAP does serve as the surrogate in driving the gene expression from genes of rickettsial organisms, including from *E. chaffeensis* promoters [29,37,44,45].

Because our prior studies demonstrated that the -35 motif, but not the -10 motif, is critical to σ^70^ binding [29], we reasoned that the -35 motif is also similarly important to σ^32^ binding. We created constructs lacking the -35 motifs from all three gene promoter segments in the plasmids containing the *dnaK*, *groE* and *hup* promoters. The modified plasmids were then used to assess for the β-galactosidase expression (Figure 5). The -35 deletions from the promoters of *dnaK* and *hup* resulted in a considerable reduction of β-galactosidase expression, whereas the enzyme activity for the *groE* promoter -35 motif deletion increased significantly compared with the wild-type promoter and the higher promoter activity was independent of the IPTG induction (Figure 5). We found a second consensus -35 motif (TTGATA) immediately upstream from the deleted -35 motif for the *groE* promoter (Figure 4A). We reasoned that repositioning the second -35 motif into the deleted first -35 motif’s location kept the spacing between the -35 and -10 motifs almost constant (16 nucleotides in the first and 17 nucleotides in the second) and that repositioning of the second motif may have functioned as a new -35 motif (Figure 4A). This secondary -35 motif, however, was not responsive to the induced expression of *E. chaffeensis* σ^32^. Complete deletion of the promoter region spanning from the first -35 motif to the entire upstream sequence of the *groE* promoter resulted in complete loss of promoter activity (Figure 5B).

### Electrophoretic Mobility Shift assays (EMSAs) to further assess the interactions of σ^32^ and σ^70^ with *E. chaffeensis* gene promoters

**Figure 6:**
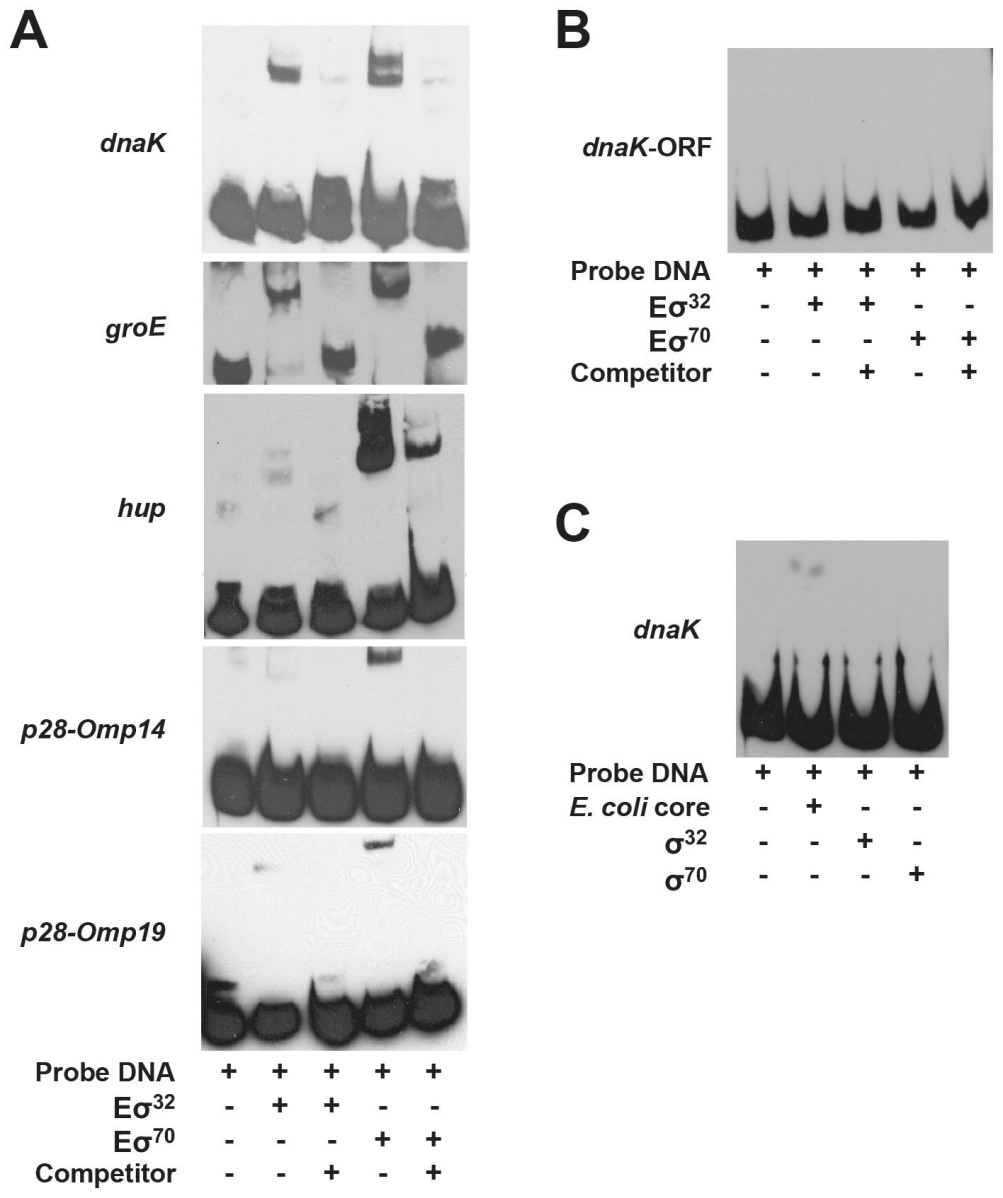
*E. chaffeensis* σ^32^ or σ^70^ binding to *dnaK*, *groE, hup*, p28-*Omp14*, p28-*Omp19* promoters assessed by EMSA analysis. A) Biotin-labeled probes of *dnaK*, *groE, hup*, p28-*Omp14* and p28-*Omp19* promoter segments were used in the EMSA analysis in the presence or absence of the RNAP holoenzyme containing either the recombinant *E. chaffeensis* σ^32^ (Eσ^32^) or σ^70^ (Eσ^70^). Specificity of RNAP binding was determined by the inclusion of 100 fold molar excess of cold competitors. B) A DNA segment containing the coding region of *dnaK* (*dnaK*-ORF) was used as a control. C) Gel shift assays were performed with *dnaK* promoter segments by incubating with *E. coli* core RNAP or purified σ^32^ or σ^70^ to serve as additional controls.

Shared recognition of *E. chaffeensis* promoters by both the transcription regulators (σ^32^ and σ^70^) (described above) validates our working hypothesis that although specificity differences exist, overlap in the recognition of -35 motifs of *E. chaffeensis* promoters by σ^32^ and σ^70^ is significant. In particular, the *dnaK* and *groE* promoters had greater specificity to the σ^32^, whereas the promoters of genes *hup, p28-Omp14* and *p28-Omp19* had higher affinity for the σ^70^. The shared recognition of *E. chaffeensis* promoters with altered specificities may have resulted due to differences in binding affinities of the sigma factors with core RNAP. To test this hypothesis, we performed EMSA analysis with probes prepared from the promoter segments of all five genes (*dnaK, groE, hup, p28-Omp14* and *p28-Omp19*) and by incubating with the RNAP holoenzyme containing *E. chaffeensis* recombinant σ^32^ or σ^70^ (Figure 6A). The promoters with higher affinity for σ^32^, *dnaK* and *groE*, had stronger gel-shifted fragments in the presence of RNAP holoenzyme with σ^32^ compared with those observed for the σ^70^ dependent promoter segments (*hup, p28-Omp14* and *p28-Omp19*). Similarly, shifted fragments were more abundant for the σ^70^-containing RNAP for the genes with higher affinity for it (*hup, p28-Omp14* and *p28-Omp19*). The specificity of gel-shifted fragments was confirmed by adding cold competitors. Specific interaction of the RNAP holoenzyme containing either σ^32^ or σ^70^ to promoter segment was further demonstrated by including additional controls; for example, holoenzyme containing σ^32^ or σ^70^ did not bind to a DNA fragment prepared from a coding sequence (*dnaK*-ORF) (Figure 6B). Similarly, σ^32^ or σ^70^ alone did not bind to a promoter segment (assessed for *dnaK* promoter) (Figure 6C). *E. coli* core enzyme, which is known to bind non-specifically to DNA [46,47], showed a minor gel shift (Figure 6C).

### Substitutions in region 4.2 of *E. chaffeensis* σ^32^

**Figure 7:**
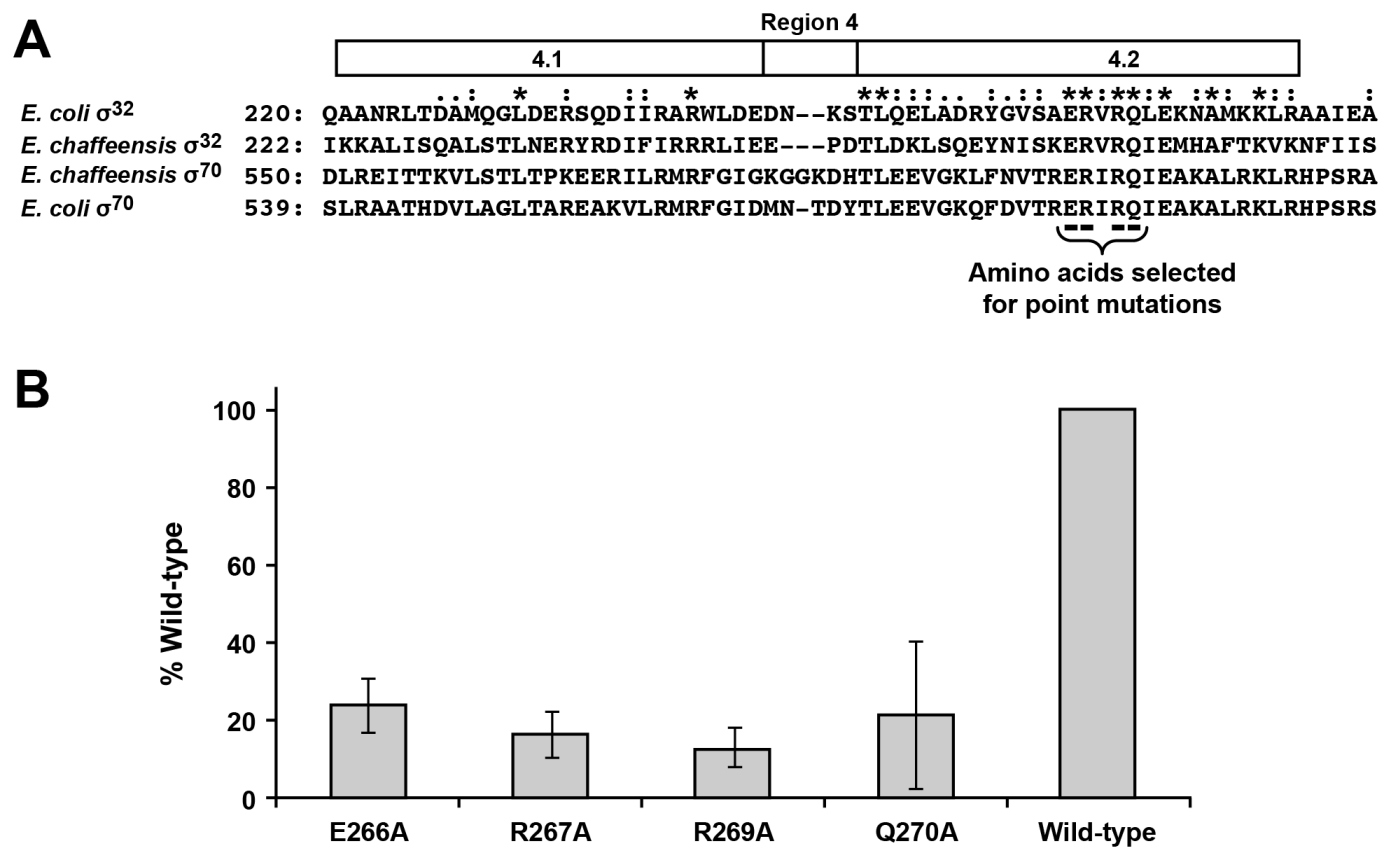
Mutational analysis of *E. chaffeensis* *rpoH* gene spanning the conserved region 4.2. A) Protein sequence homology of σ^32^ and σ^70^ of *E. chaffeensis* and *E. coli* was assessed by Clustal X (version 2.0) for the entire sequence. The homology spanning region 4 was presented here. Numbers on the left indicate the amino acid position relative to the start codon of each protein. The four amino acids in *E. coli*, which are identified as critical for binding to the -35 motifs of σ^32^ and σ^70^, are also conserved in *E. chaffeensis* (highlighted with an underlined text). Amino acids that are conserved in all four protein primary sequences are identified with asterisks; homology found only in two or three proteins was identified with a dot or colon, respectively. B) Mutational analysis of the four conserved amino acid residues predicted to be involved in the binding of σ^32^ to the -35 motif. The amino acids at positions E266, R267, R269 and Q270 of *E. chaffeensis* σ^32^ were individually mutated to change the amino acids in the encoded proteins each to alanine. The mutant plasmids were used to assess the *E. chaffeensis* σ^32^ in driving the promoter activity of the wild-type *dnaK* gene (β-galactosidase expression measured relative to the wild-type *E. chaffeensis* σ^32^). The experiment was performed three times, and average values were presented with error bars to show the standard deviation.

Previous research in *E. coli* demonstrated that four conserved, charged amino acids within the region 4.2 of σ^32^, E265, R266, R268 and Q269, are essential for binding to the -35 motif of a promoter sequence [48]. To determine if the amino acids are similarly conserved, sequence alignment was performed for *E. chaffeensis* σ^32^ and σ^70^ with those of *E. coli*, which revealed extensive amino acid conservation among all four sequences. The conserved amino acids indeed included the four charged amino acids of the region 4.2 (Figure 7A). To assess if mutations in these four amino acids in *E. chaffeensis* gene similarly affect the promoter recognition, individual substitution mutations were made in the recombinant plasmid sequences spanning the four amino acids in the *E. chaffeensis rpoH* gene coding sequence and were used to express mutant proteins and their impact on the β-galactosidase expression driven from the wild-type *dnaK* promoter. Amino acid changes at each of the four locations to the non-polar amino acid (alanine) resulted in about an 80% decline in transcription activity (assessed by β-galactosidase expression) compared with that observed for wild-type σ^32^ (Figure 7B).

### Expression patterns of *E. chaffeensis* rpoD and rpoH gene transcripts during the infection of mammalian cells are very similar

**Figure 8:**
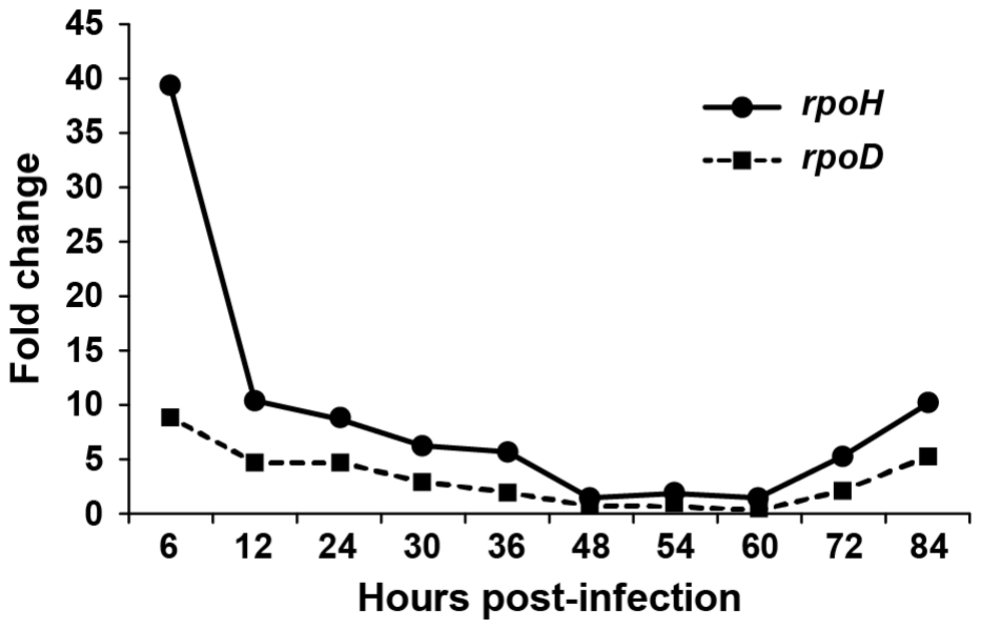
Transcripts of σ^32^ and σ^70^ assessed in *E. chaffeensis* infected macrophages. Total RNA recovered from *E. chaffeensis* infected macrophages at different times post-infection was assessed by quantitative RT-PCR after normalizing the RNA levels used for the analysis relative to 16S rRNA and the data were presented as the fold change at each time point post-infection relative to zero time point. The experiment was performed three times, and the average values were used for plotting the graph.

To assess the expression patterns of *rpoH* (σ^32^ gene) and *rpoD* (σ^70^ gene), *E. chaffeensis* RNA isolated from infected macrophage cultures at different times post infection was examined by TaqMan probe-based quantitative RT-PCR assay (Figure 8). The expression levels of both *rpoD* and *rpoH* were initially high following inoculation, with relatively higher expression from the *rpoH* gene at 6 h post-infection. Both the gene transcripts decreased steadily, however, and remained low until 60 h post infection and slightly increased thereafter. No notable differences were found in the expression patterns for the *rpoD* and *rpoH* gene transcripts during the 84 h of assessment of the organism’s growth in macrophage cultures.

## Discussion

Life for an intraphagosomal bacterium is complex because the organisms must adapt to the host’s phagosomal micro-environment, which is suboptimal for bacterial growth. The intraphagosomal bacterium, *E. chaffeensis*, has an unusual developmental cycle requiring the growth and replication within phagosomes of eukaryotic cells of vertebrate and tick hosts. During its developmental cycle, *E. chaffeensis* exists in two distinct morphological forms: the elementary bodies (EBs) and the reticulate bodies (RBs) [49-51]. EB (the infectious form) transforms to metabolically active RB after entry into a host cell and replicates by binary fission [49-51]. Very little is known about how the organism overcomes the host-induced stress in support of its invasion and replication in host phagosomes and its subsequent release and reinfection of naïve host cells. Cheng et al. [52] recently presented the first evidence of the possible involvement of a response regulator, CtrA, of *E. chaffeensis* in human monocytes when the organism develops into EBs. The CtrA binds to the promoter regions of several genes activated during this stage of development; however, much remains to be understood about how *E. chaffeensis* regulates its gene expression in support of its growth in a host cell. Growth of the organism in vertebrate and tick host cells results in altering expression of many genes; recent studies revealed many host-specific differences in gene expression as assessed by global changes in gene and protein expression [13,14]. It is entirely unknown how the organism senses the host environment and alters its gene expression. One possibility is that the differential expression may be accomplished by regulating gene expression by DNA binding proteins which influence the function of RNAP. This hypothesis remains to be tested.

Host adaptation of a bacterium requires altering its gene expression of many genes, including those encoding for stress response proteins and many outer membrane proteins. For example, the heat shock protein ClpB is an ATP-dependent molecular chaperone that reactivates aggregated proteins accumulating under stress conditions [53]. We recently presented the first evidence that *clpB* gene expression increases during replication stage of *E. chaffeensis* [54]. Similarly, we and others reported global macrophage and tick cell-specific protein and gene expression differences [13,14]; the differentially expressed proteins included various outer membrane proteins [11,13,55,56]. 

The sigma factor, σ^32^, is considered the chief regulatory protein of transcription of genes under stressful environments in bacteria, and genes expressed with the help of this regulator include many conserved heat-shock proteins that help bacteria resist stress by decreasing the accumulation of misfolded and aggregated proteins [57-59]. On the contrary, σ^70^ is considered the primary housekeeping sigma factor [20]. Considerable progress has been made in defining the contributions of alternate sigma factor (σ^32^) in support of stress response in *E. coli* [60]. Recent study on *Francisella tularensis* demonstrates that σ^32^ also contributes to the stress response in this intracellular pathogenic bacterium [61]; however, research is limited about understanding the role of sigma factors in intraphagosomal bacteria such as *E. chaffeensis* and other related rickettsiae organisms. Knowledge about how regulation of gene expression is accomplished by the pathogenic rickettsiae is similarly limited. In this study, we utilized multiple molecular tools to assess the functions of the only two sigma factors (σ^32^ and σ^70^) of *E. chaffeensis*. We mapped the transcription start sites of 12 genes of *E. chaffeensis*, which are likely recognized by the pathogen’s σ^32^ or σ^70^ (10 in the current study and two reported previously [29]). We examined the promoter activities of several *E. chaffeensis* genes *in vitro* using the *E. coli* expression system. We discovered that the first three nucleotides are identical for both the -10 and -35 motifs in the predicted σ^32^ and σ^70^ binding motifs (-10 and -35 motifs) of *E. chaffeensis* gene promoters; we presented evidence supporting the shared recognition of *E. chaffeensis* gene promoters by both σ^32^ and σ^70^, although the specificities of promoters for each sigma factor are different. For example, promoters of the predicted stress-response genes, *groE* and *dnaK*, are primarily recognized by the alternate sigma factor, σ^32^, whereas the *hup*, *p28-Omp14* and *p28-Omp19* gene promoters had higher affinity to σ^70^. Interestingly, the host cell-specific differentially expressed membrane protein genes, *p28-Omp14* and *p28-Omp19*, although predominantly transcribed by σ^70^, are also transcribed by the pathogen’s likely alternate sigma factor, σ^32^, at a relatively high rate. The shared recognition of the pathogen gene promoters by RNAP containing either σ^32^ or σ^70^ in transcribing genes of *E. chaffeensis* is intriguing and suggests that its transcription system is evolved to be co-regulated by both sigma factors in controlling the gene expression. Our transcriptional analysis of the *E. chaffeensis rpoH* and *rpoD* genes revealed similar expression patterns of an initial burst during the first 6 h post-infection, then maintaining expression with a slow decrease in both transcripts from 12 h to 60 h post-infection, then a minor increase in transcription from 60-84 h post-infection. The observed changes in the gene expression of *rpoD* and *rpoH* probably parallels the organism’s transformation to RBs, their continued replication and then reversion into EBs [1]. The transcription regulators, σ^32^ or σ^70^, in *E. chaffeensis* may function as a team in regulating gene expression. Gene regulation in *E. chaffeensis* may also involve the contributions of transcription regulatory proteins, specifically to alter gene expression in support of adapting the organism to dual host environments. Progress described here in understanding gene regulation is the important first step, and continued research in this virtually unexplored territory is warranted.

In proteobacteria, regardless of α, β, γ and δ subdivision, the -35 motif of the σ^32^-dependent genes shows the sequence conservation of the first three residues as ‘TTG’ [61-67]. The consensus -35 motifs in all assessed *E. chaffeensis* genes, independent of their prediction as the σ^32^ or σ^70^ dependent genes, included the same three conserved nucleotides. In this study, we presented evidence that the deletion of the predicted -35 motifs of *dnaK*, *groE* and *hup* promoters significantly decreased promoter activities. We reported earlier that the deletion of -35 motifs in *p28-Omp14* and *p28-Omp19* gene promoters also results in a significant decline in promoter activity [29]. Experimental data presented here demonstrated that the binding of σ^32^ to -35 motif of a promoter is mediated by the four well-conserved charged amino acids in region 4.2. Sigma factors are composed of a variable number of regions, each having a specific function in promoter recognition and region 4.2 in *E. coli* σ^32^ is shown to be involved in the base-specific interaction with the -35 motif. In particular, mutations causing substitutions in four conserved charged amino acids at E265, R266, R268 and Q269 to a non-polar amino acid, alanine, significantly reduce the promoter activity of *E. coli* σ^32^ [48]. Because these four amino acids are also conserved in *E. chaffeensis* σ^32^ and σ^70^, mutational analysis in modifying the corresponding four conserved amino acids in the *rpoH* gene of *E. chaffeensis* to alanine similarly reduced the promoter activity of its *dnaK* promoter. Together these results demonstrate that the -35 motifs are important to recognition of gene promoters by sigma factors in *E. chaffeensis*. 

Considering the shared recognition of *E. chaffeensis* promoters by RNAP containing either σ^32^ or σ^70^ in transcribing the gene products, the pathogen’s gene regulation may have evolved requiring the interplay of both σ^32^ and σ^70^ and DNA binding proteins (transcription regulators) in support of its continued survival in vertebrate and tick hosts and in altering host cell-specific gene expression. This hypothesis, however, remains to be tested. We expect this study to lead the way in furthering our understanding of the regulation of gene expression in *E. chaffeensis*. This study also will aid future research in defining the molecular mechanisms underlying the adaptation of *E. chaffeensis* to the host cell environment.

## Conclusions

This study is the first to utilize various molecular approaches as useful in defining the promoters of *E. chaffeensis* genes. We mapped the transcription start sites of 8 *E. chaffeensis* genes and evaluated promoters of five genes; *groE, dnaK, hup, p28-Omp14* and *p28-Omp19* genes. We also presented evidence that the RNA polymerase binding motifs of *E. chaffeensis* gene promoters are highly homologous for its only two transcription regulators, sigma 32 and sigma 70, and that gene expression is accomplished by either of the transcription regulators. Evidence was also presented demonstrating that the sigma 32 and sigma 70 mRNAs are upregulated during the early replicative stage of the organism and the expression patterns remained similar for the entire replication cycle. Evidence was also presented demonstrating that the organism’s -35 motifs are essential to transcription initiations.

## Supporting Information

Table S1 **Oligonucleotides use in this study.**
(DOCX)
