# Supplementary material for: Transcription of Ehrlichia chaffeensis Genes Is Accomplished by RNA Polymerase Holoenzyme Containing either Sigma 32 or Sigma 70
Source: PLoS One. 2013 Nov 21;8(11):e81780. doi: 10.1371/journal.pone.0081780 (PMC3836757; doi:10.1371/journal.pone.0081780)
Supplement: Table S1 — Oligonucleotides use in this study. (DOCX) [file pone.0081780.s001.docx]

Table S1: Oligonucleotides use in this study

| Number | Sequences | Orientation | | | Use | | |  |  |  |  |  |
| --- | --- | --- | --- | --- | --- | --- | --- | --- | --- | --- | --- | --- |
| For primer extension (PE) and for cloning promoter segments (CPS) used as templates of sequencing | | | | | | | | | | | | |
| RRG812 | 5’-taacttacttaaaaaattaactacttaaatatc-3’ | | | Forward | CPS for *groE* | | | | |  |  |  |
| RRG808 | 5’-ctgaatcaggaagctgtatagg-3’ | | | Reverse | CPS and PE for *groE* | | | | |  |  |  |
| RRG815 | 5’-actactattaattttcttgaa-3’ | | | Forward | CPS for *hup* | | | | |  |  |  |
| RRG811 | 5’-ttgtgtaacctaattagatgagaag-3’ | | | Reverse | CPS and PE for *hup* | | | | |  |  |  |
| RRG1108 | 5’-tcatagctagtatagctgatgctcca-3’ | | | Forward | CPS for *clpA* | | | | |  |  |  |
| RRG1109 | 5’-agatgttccaaagtagcatactcatg-3’ | | | Reverse | CPS and PE for *clpA* | | | | |  |  |  |
| RRG1104 | 5’-ctacacaataacatcataacctacgtgag-3’ | | | Forward | CPS for *hslV* | | | | |  |  |  |
| RRG1103 | 5’-attacagtgtgtcctaacgatacttgc-3’ | | | Reverse | CPS and PE for *hslV* | | | | |  |  |  |
| RRG1099 | 5’-acagtaccctatcatcttttgatattcag-3’ | | | Forward | CPS for *clpB* | | | | |  |  |  |
| RRG1100 | 5’-ctaacattactttaagtaaatgctctgg-3’ | | | Reverse | CPS and PE for *clpB* | | | | |  |  |  |
| RRG816 | 5’-atgttgtagtgttaggttgtaatagt-3’ | | | Forward | CPS for *DNAbp* | | | | |  |  |  |
| RRG817 | 5’-tatatcatatacaaaagacttaccaac-3’ | | | Reverse | CPS and PE for *DNAbp* | | | | |  |  |  |
| RRG820 | 5’-taattcataaatttatgaacttgtg-3’ | | | Forward | CPS for *dnaK* | | | | |  |  |  |
| RRG821 | 5’-tgcaactattgaaggtgtagttc-3’ | | | Reverse | CPS and PE for *dnaK* | | | | |  |  |  |
| For use in 5’ RACE experiments | | | | | | | | | | | |  |
| RRG1232 | 5’-ctccataccattacaggttacttc-3’ | | | Reverse | *glyQ* | | | |  |  |  |  |
| RRG1231 | 5’-caatgcttggattttcccaatcgtcttc-3’ | | | Reverse | *glyQ* (nested PCR) | | | |  |  |  |  |
| RRG1234 | 5’-tcttttctcctgtttcctcaca-3’ | | | Reverse | *dksA* | | | |  |  |  |  |
| RRG1233 | 5’-tctgcatcagcttgtggacgtagt-3’ | | | Reverse | *dksA* (nested PCR) | | | |  |  |  |  |
| RRG1078 | 5’-tctagtgatgttgtgacgttctagagtg-3’ | | | Reverse | *grpE* | | | |  |  |  |  |
| RRG1077 | 5’-atcagtactcctgcatgaatactgtcac-3’ | | | Reverse | *grpE* (nested PCR) | | | |  |  |  |  |
| For cloning *E. chaffeensis* *rpoH* into pET32 plasmid* | | | | | | | | | | |  |  |
| RRG744 | 5’-GAGCCATGGctttaacaaattctatattttccctaactc-3’ | | Forward | | | |  | | | |  |  |
| RRG745 | 5’-CGACTCGAGttaactattgatattacaatgacctagt-3’ | Reverse | | | |  | | | | |  |  |

Supplemental Table 1 (continued)

| Number | Sequences | Orientation | | Use | | |  |
| --- | --- | --- | --- | --- | --- | --- | --- |
| For cloning *E. chaffeensis* promoters into pMT504 plasmid | | | | | | | |
| RRG1203 | 5’-cacaacatttaaagtgttgttattacc-3’ | | Forward | *hup* | |  |  |
| RRG1301 | 5’-taattttagataagtgaagaagatttagg-3’ | | Reverse | *hup* | |  |  |
| RRG1205 | 5’-aacgatgttttcagtatcaagtc-3’ | | Forward | *groE* | |  |  |
| RRG1207 | 5’-taagatcatatataatatcaagaaatct-3’ | | Reverse | *groE* | |  |  |
| RRG862 | 5’-aggatgctgttaatgtatttactgttg-3’ | | Forward | *dnaK* | |  |  |
| RRG863 | 5’-tataatcacagatataataaccaaatc-3’ | | Reverse | *dnaK* | |  |  |
| RRG217^$^ | 5’-attgctcaaccataaaataatggga-3’ | | Forward | *p28-Omp14-35* | |  |  |
| RRG695^^^ | 5’-taaaaatttaagaataatgaaag-3’ | | Reverse | *p28-Omp14-35* | |  |  |
| RRG185^$^ | 5’-gactctagacttttaattttattattgccacatg-3’ | | Forward | *p28-Omp19-35* | |  |  |
| RRG696^^^ | 5’-aaataaattaacaatagtagaag-3’ | | Reverse | *p28-Omp19-35* | |  |  |
| For cloning *E. chaffeensis* *rpoH* into pSAKT32 plasmid | | | | | | | |
| RRG933 | 5’-gtcgtcgacctttaagaaggagatataccatgttaacaaattctatattttccctaac-3’ | | Forward | |  | | |
| RRG932 | 5’-agcacaaaaaaaagcccgctcattaggcgggctggcattagggtatttgtatagatgc-3’ | | Reverse | |  | | |
| For cloning *E. chaffeensis* promoters into pQF50K plasmid* | | | | | | | |
| RRG951 | 5’-CAGCGCATGCcataaatttatgaacttgtgttaaatg-3’ | | Forward | *dnaK* | |  |  |
| RRG952 | 5’-GCTGTCTAGAttaactataatcacagatataataacc-3’ | | Reverse | *dnaK* | |  |  |
| RRG955 | 5’-CAGCGCATGCgcagattaattaataatgctttaag-3’ | | Forward | *hup* | |  |  |
| RRG956 | 5’-GCTGTCTAGAttcataactgaacaagatttaggatattag-3’ | | Reverse | *hup* | |  |  |
| RRG969 | 5’-CAGCGCATGCgatttcttgatattatatatgatcttagc-3’ | | Forward | *groE-35updel* | |  |  |
| RRG970 | 5’-GCTGTCTAGAtacctctataaaaaaataaattatcagtag-3’ | | Reverse | *groE-35updel* | |  |  |
| RRG1105 | 5’-CAGCGCATGCtaacttacttaaaaaattaactacttaaatatc-3’ | | Forward | *groE* | |  |  |
| RRG970 | 5’-GCTGTCTAGAtacctctataaaaaaataaattatcagtag-3’ | | Reverse | *groE* | |  |  |
|  |  | |  |  | |  |  |

Supplemental Table 1 (continued)

| Number | Sequences | Orientation | | Use |  |  |  |
| --- | --- | --- | --- | --- | --- | --- | --- |
| For -35 motif deletion of *E. chaffeensis* promoters in pQF50K | |  |  | | | |  |
| RRG1155 | 5’-cataaatttatgaacttgtgttaaatggggtcttatgatttggttattatatctgtgatt-3’ | Forward | *dnaK* | | | |  |
| RRG1161 | 5’-aatgctttaagtaataacaaataaatttagatatgaactaacctaatatcctaaatcttg-3’ | Forward | *hup* | | | |  |
| RRG1163 | 5’-gtgcaatatgtattataaaatttattgattgatacttagatttcttgatattatatatgatcttagctag-3’ | Forward | *groE* | | | |  |
|  |  |  |  | | | |  |
| For EMSA probe or competitor | |  |  | | | |  |
| RRG1480 | 5’-Biotin-taacttacttaaaaaattaactactta-3’ | Forward | *groE* probe | | | |  |
| RRG1105 | 5’-cagcgcatgctaacttacttaaaaaattaactacttaaatatc-3’ | Forward | *groE* competitor | | | |  |
| RRG1458 | 5’-ctacctctataaaaaaataaattatcagtagcatc-3’ | Reverse | *groE* probe/competitor | | | |  |
| RRG1459 | 5’-Biotin-ttcataaatttatgaacttgtgttaaat-3’ | Forward | *dnaK* probe | | | |  |
| RRG951 | 5’-cagcgcatgccataaatttatgaacttgtgttaaatg-3’ | Forward | *dnaK* competitor | | | |  |
| RRG1493 | 5’-gtacctaaatctatacctataacag-3’ | Reverse | *dnaK* probe/competitor | | | |  |
| RRG1544 | 5’-Biotin-agtattcttgctggtgatgttagag-3’ | Forward | *dnaK*-ORF probe | | | |  |
| RRG1549 | 5’-agtattcttgctggtgatgttagag-3’ | Forward | *dnaK*-ORF competitor | | | |  |
| RRG1548 | 5’-catgtactataccattagcatctatgtc-3’ | Reverse | *dnaK*-ORF probe/competitor | | | |  |
| RRG1461 | 5’-Biotin-attgcagattaattaataatgctttaag-3’ | Forward | *hup* probe | | | |  |
| RRG955 | 5’-cagcgcatgcgcagattaattaataatgctttaag-3’ | Forward | *hup* competitor | | | |  |
| RRG1462 | 5’-taattttaactcctaattttacataactgaacaagatttaggata-3’ | Reverse | *hup* probe/competitor | | | |  |
| RRG1463 | 5’-Biotin-ctgacgtaatatattaaattttccttac-3’ | Forward | *p28-Omp19* probe | | | |  |
| RRG277^$^ | 5’-ctgacgtaatatattaaattttcc-3’ | Forward | *p28-Omp19* competitor | | | |  |
| RRG1464 | 5’-tgtaattcatatataacctaatagtgacaaataaatta-3’ | Reverse | *p28-Omp19* probe/competitor | | | |  |
| RRG1465 | 5’-Biotin-aagcaagtctactcatatttttatta-3’ | Forward | *p28-Omp14* probe | | | |  |
| RRG1294 | 5’-aagtggtaaaagcaagtctactcatatttttatta-3’ | Forward | *p28-Omp14* competitor | | | |  |
| RRG1466 | 5’-catgttaataaaccttttataaaagataata-3’ | Reverse | *p28-Omp14* probe/competitor | | | |  |
| For substitution in 4.2 region of *E. chaffeensis* σ^32#^ | | | | | | | |
| RRG1270 | 5’-agtcaagagtataatatatcaaaag**C**gagagttagacaaatagaaatgcatgcttttact-3’ | Forward | Substitution E266A | | |  |  |
| RRG1271 | 5’-agtcaagagtataatatatcaaaagag**GC**agttagacaaatagaaatgcatgcttttact-3’ | Forward | Substitution R267A | | |  |  |
| RRG1272 | 5’-agtcaagagtataatatatcaaaagagagagtt**GC**acaaatagaaatgcatgcttttact-3’ | Forward | Substitution R269A | | |  |  |
| RRG1273 | 5’-agtcaagagtataatatatcaaaagagagagttaga**GC**aatagaaatgcatgcttttact-3’ | Forward | Substitution Q270A | | |  |  |

| Number | Sequences | Orientation | Use |
| --- | --- | --- | --- |
| For expression patterns of *E. chaffeensis* *rpoD* and *rpoH* genes | |  |  |
|  |  |  |  |
| RRG759 | 5’-gaagatgatgtatcagacacc-3’ | Forward | *rpoD* RT-PCR |
| RRG760 | 5’-ttcacgagatagtaattcaacag-3’ | Reverse | *rpoD* RT-PCR |
| RRG761 | 5’-cttgggattttggacaaacagatgaccct-3’ | probe | TaqMan probe for *rpoD* |
| RRG762 | 5’-ctcctgaagaagaagacagg-3’ | Forward | *rpoH* RT-PCR |
| RRG763 | 5’-gactactagccttagatgactag-3’ | Reverse | *rpoH* RT-PCR |
| RRG764 | 5’-atgggatcgttgctgacgcacataggtt-3’ | probe | TaqMan probe for *rpoH* |

Supplemental Table 1 (continued)

* Capital letters with underline refer to sequences inserted for creating restriction enzyme sites. Three or four nucleotides were also included in the primers at the 5’ end to facilitate restriction enzyme site accessibility by restriction enzymes.

# Bold capital letters refer to the change of a base for creating substitution of an amino acid.

$ These primers were described earlier as in [29].

^ These primers were described earlier as in [37].
